# Oyster Mushroom Spherical Virus Crosses the Species Barrier and Is Pathogenic to a New Host *Pleurotus pulmonarius*

**DOI:** 10.3390/ijms241310584

**Published:** 2023-06-24

**Authors:** Xiaoyan Zhang, Haijing Hu, Yanxiang Zhao, Yifan Wang, Wenjing Zhang, Lunhe You, Jianrui Wang, Yu Liu, Xianhao Cheng

**Affiliations:** 1 School of Agriculture, Ludong University, Yantai 264025, China; xiaoyan433@163.com (X.Z.); haijing975@163.com (H.H.); wangyifan7798@126.com (Y.W.); 17862283938@163.com (W.Z.); youlunhe98@163.com (L.Y.); jianrui302@163.com (J.W.); 2 College of Plant Health and Medicine, Key Lab of Integrated Crop Disease and Pest Management of Shandong Province, Qingdao Agricultural University, Qingdao 266109, China; zhaoyx@qau.edu.cn

**Keywords:** oyster mushroom spherical virus, *Pleurotus pulmonarius*, co-culturing, horizontal transmission

## Abstract

Oyster mushroom spherical virus (OMSV) is a mycovirus with a positive-sense single-stranded RNA genome that infects the edible mushroom *Pleurotus ostreatus*. OMSV is horizontally transferred from an infected strain to a cured strain via mycelia. The infection results in significant inhibition of mycelial growth, malformation of fruiting bodies, and yield loss in oyster mushrooms. This study successfully transferred OMSV from *P. ostreatus* to *Pleurotus pulmonarius*. However, transmission was not successful in other *Pleurotus* species including *P. citrinopileatus*, *P. eryngii*, *P. nebrodensis*, and *P. salmoneostramineus*. The successful OMSV infection in *P. pulmonarius* was further verified with Western blot analysis using a newly prepared polyclonal antiserum against the OMSV coat protein. Furthermore, OMSV infection reduced the mycelial growth rate of *P. pulmonarius*. The OMSV-infected strain demonstrated abnormal performance including twisted mushrooms or irregular edge of the cap as well as reduced yield of fruiting bodies in *P. pulmonarius*, compared to the OMSV-free strain. This study is the first report on the infection and pathogenicity of OMSV to the new host *P. pulmonarius*. The data from this study therefore suggest that OMSV is a potential threat to *P. pulmonarius*.

## 1. Introduction

Mycoviruses are widespread in most fungi, including edible fungal species [1]. Most mycoviruses contain double-stranded RNA (dsRNA) genomes, while some have single-stranded RNA (ssRNA), single-stranded DNA (ssDNA), or double-stranded DNA (dsDNA) genomes [2]. In many cases, mycovirus alter the host fungus phenotype. Mutualism is also common between mycoviruses and their hosts [3,4]. Many edible mushroom species are infected with mycoviruses, including *Agaricus bisporus*, *Lentinula edodes*, *Pleurotus ostreatus*, *Flammulina velutipes*, *Pleurotus eryngii*, *Agrocybe aegerita*, *Boletus edulis*, *Volvariella volvacea*, *Grifola frondose*, *Armillaria* species., *Auricularia heimuer*, *Bondarzewia berkeleyi*, *Picoa juniperi*, *Leucocybe candicans*, *Cordyceps chanhua*, and *Pleurotus citrinopileatus* [1,5,6,7,8,9,10,11,12,13,14,15,16,17,18,19]. These mycoviruses can enhance or diminish various symptoms in the host fungi, including changes in morphology, sporulation, pigmentation, radial growth, and/or biomass production [20,21].

Mycoviruses lack an extracellular phase for invading new hosts, as they are usually horizontally transmitted via hyphal anastomosis or vertically via asexual and sexual spores in nature [22]. Mycoviruses can only infect the same or closely related vegetative compatibility groups [23]. However, some mycoviruses can be vertically transmitted via basidiospores such as La France isometric virus (LIV) in *A. bisporus*, and in *L. edodes,* the Lentinula edodes negative-stranded RNA virus 1 (LeNSRV1), Lentinula edodes spherical virus (LeSV), and Lentinula edodes partitivirus 1 (LePV1) [6,24,25,26]. In the laboratory, *Cryphonectria parasitica* and other *Cryphonectria* species were co-cultured, and a very strong vegetative incompatibility barrage was detected between the colonies. However, six out of the ten *Cryphonectria* species isolates tested were successfully infected by Cryphonectria hypovirus 1 (CHV-1) from *C. parasitica* via hyphal anastomosis [27]. Cornejo et al. (2021) demonstrated that the Cryphonectria naterciae fusagravirus 1 (CnFGV1) can spread not only within species but also via cross-species transmission. CnFGV1 can be easily transmitted in *Cryphonectria. naterciae* via asexual spores to the next generation of the experimentally-infected *Cryphonectria* species [28]. In *A. bisporus*, the mushroom virus X (MVX) can spread horizontally via hyphal anastomosis from an infected strain to five other virus-free strains [29]. Recently, a novel mycovirus Cordyceps chanhua partitivirus 1 (CchPV1) in *C. chanhua* was reported to be transmitted horizontally from strain RCEF5997 to strain RCEF5833 [18]. In our previous studies, OMSV was transmitted horizontally from an OMSV-infected strain to the cured strain [30]. Therefore, further investigation of the cross-species virus transmission of OMSV was necessary.

*Pleurotus ostreatus* (oyster mushroom) is a widely cultivated edible saprophytic basidiomycete [31]. Several mycoviruses infecting this mushroom have been previously identified, including the dsRNA viruses Pleurotus ostreatus virus 1 (PoV1), Pleurotus ostreatus spherical virus (POSV), oyster mushroom isometric virus (OMIV), Pleurotus ostreatus virus ASI2792 (PoV-ASI2792), Pleurotus ostreatus virus Shin-Nong (PoV-SN), and the only positive-sense (+) ssRNA virus, oyster mushroom spherical virus (OMSV) [5,32,33,34,35,36]. OMSV is closely related to the mushroom die-back disease and first was detected in Korea infecting *P. ostreatus* mushroom crops in 2003. The spherical virion is 27 nm in diameter and has a genome size of 5784 nucleotides with seven open reading frames (ORFs). The RNA-dependent RNA polymerase (RdRP) and coat protein (CP) are encoded by ORF1 and 2, respectively [5]. Previously, an OMSV-China strain was identified from the *P. ostreatus* 8129 strain, which negatively affects mycelial growth, distorts fruiting bodies, and decreases mushroom yield [30].

This study aimed to identify if OMSV could infect and threaten other *Pleurotus* species. Co-cultivation assays demonstrated that OMSV can horizontally transfer from *P. ostreatus* to *P. pulmonarius* but not to *P. eryngii*, *P. citrinopileatus*, *P. nebrodensis*, or *P. salmoneostramineus*. Furthermore, OMSV infection reduced the mycelial growth rate of *P. pulmonarius,* and the OMSV-infected mushrooms displayed abnormal performance. Moreover, the OMSV infection reduced the yield of fruiting bodies in *P. pulmonarius*. The current study is the first to report the details of the OMSV infection in the new host *P. pulmonarius*. The results of this study demonstrate that OMSV is a potential threat to *P. pulmonarius* and possibly to other *Pleurotus* species.

## 2. Results

### 2.1. OMSV Infects the New Host Pleurotus pulmonarius across the Species Barrier

**Figure 1:**
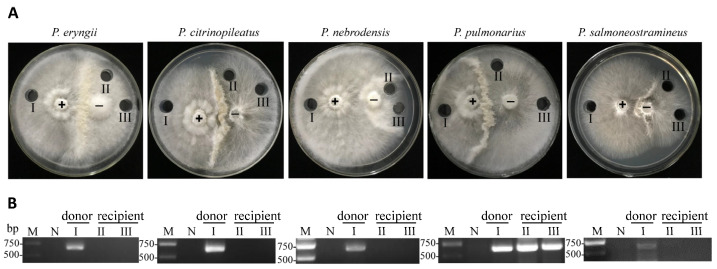
Co-cultivation of OMSV-positive (+) *Pleurotus ostreatus* and OMSV-free (−) *Pleurotus* spp. (**A**) Co-culture of the donor strain with replicate C1021 of *P. eryngii*, replicate Y055 of *P. citrinopileatus*, replicate BN18 of *P. nebrodensis*, replicate XH2208 of *P. pulmonarius*, and replicate TH20901 of *P. salmoneostramineus*. (**B**) RT-PCR detection of OMSV in different inocula. OMSV-free strains were used as negative controls (N). M, DNA Marker2000.

Previous research has shown that OMSV can horizontally transfer to a virus-cured isogenic strain [30]. In order to test if the OMSV transmission could occur between different *Pleurotus* species, we collected the strains of *P. eryngii*, *P. citrinopileatus*, *P. nebrodensis*, *P. pulmonarius*, and *P. salmoneostramineus*. By the multiplex RT-PCR detection, all the stains were negative for OMSV, OMIV, POSV, or PoV1 (Figure S1). Then, the OMSV-infected *P. ostreatus* 8129 strain was used as a donor and co-cultivated with the five recipient strains on potato dextrose agar (PDA) plates. Following several days of co-cultivation, a strong and clear barrage line between donor and recipient strains was observed (Figure 1A). One inoculum from the region of the donor culture and two from the recipient culture were then sub-cultivated. After seven days of culture, the presence or absence of OMSV in all strains was detected using reverse transcription (RT)-PCR. OMSV was positive only in the *P. pulmonarius* and negative in the other four *Pleurotus* species under the same conditions (Figure 1B). Thus, the results suggest that the OMSV was successfully transferred from *P. ostreatus* to *P. pulmonarius*.

**Figure 2:**
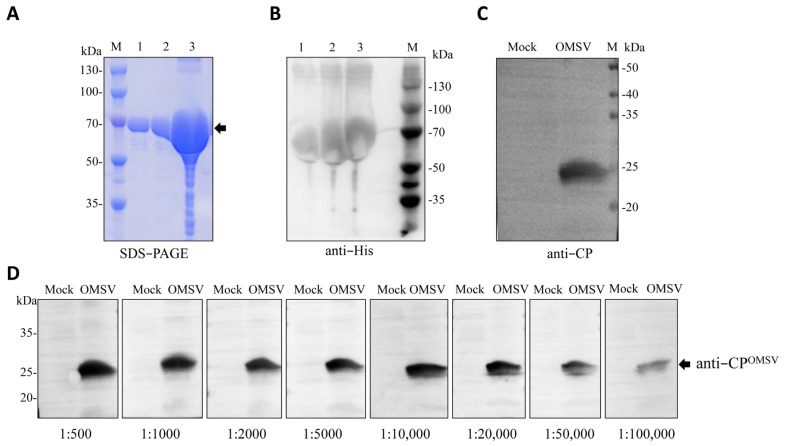
Prokaryotic expression and purification of OMSV-CP recombinant protein and preparation of polyclonal antiserum. (**A**) The purified His.MBP-OMSV-CP recombinant protein in SDS-PAGE and (**B**) Western blot. Lane M, molecular weights of protein marker; lanes 1–3, purified recombinant protein with concentrations of 0.5, 1.0, and 5.0 mg/mL, respectively. The arrow indicated the recombinant protein. (**C**) Specificity analysis of OMSV-CP antiserum using Western blotting. The left lane shows the negative control, and the right contains proteins extracted from OMSV-infected *P. pulmonarius* mycelia. (**D**) Titer determination of OMSV-CP antiserum. The antiserum was used at eight different dilutions (1:500, 1:1000, 1:2000, 1:5000, 1:10,000, 1:20,000, 1:50,000, and 1:100,000) against OMSV. The bands corresponding to the samples in (**C**) are presented.

To further confirm that OMSV can stably replicate in *P. pulmonarius*, polyclonal antiserum against OMSV-CP was prepared. The amplified OMSV *CP* gene was subcloned into the pDB.His.MBP vector to obtain the pDB.His.MBP-CP^OMSV^ recombinant vector. The predicted His-MBP-CP^OMSV^ fusion protein was 67 kDa in size. After prokaryotic expression and purification, sodium dodecyl sulfate–polyacrylamide gel (SDS-PAGE) electrophoresis showed a clear band near the 70 kDa protein marker (Figure 2A). Western blot analysis with His-tag rabbit polyclonal antibody demonstrated the presence of the signal at the same position (Figure 2B), suggesting that the MBP-CP^OMSV^ fusion protein was successfully purified. The specific antiserum against CP^OMSV^ was obtained via rabbit immunization using the purified protein. To further investigate if the antiserum was OMSV specific, the total protein extracts of the OMSV-free and OMSV-infected *P. pulmonarius* mycelia were used for Western blotting using the CP^OMSV^ polyclonal antibody. The antiserum was positive in reactions with OMSV-infected *P. pulmonarius* mycelia which had a specific protein band at approximately 24 kDa, but no reaction was observed in the OMSV-free strain (Figure 2C). These results suggest that the developed antiserum effectively detected the OMSV. The titer of the CP^OMSV^ antiserum was then detected using Western blotting with OMSV-infected *P. pulmonarius* mycelia at the ratio range from 1:500 to 1:100,000 (Figure 2D). A clear protein band of 24 kDa was detected down to a dilution of 1:100,000, suggesting that the prepared OMSV CP antiserum displayed high sensitivity.

Taken together, RT-PCR and serological detection co-cultivation tests confirmed that OMSV can persistently infect *P. pulmonarius* across the incompatibility barrier.

### 2.2. OMSV Infection Slows the Mycelial Growth Rate of P. pulmonarius

**Figure 3:**
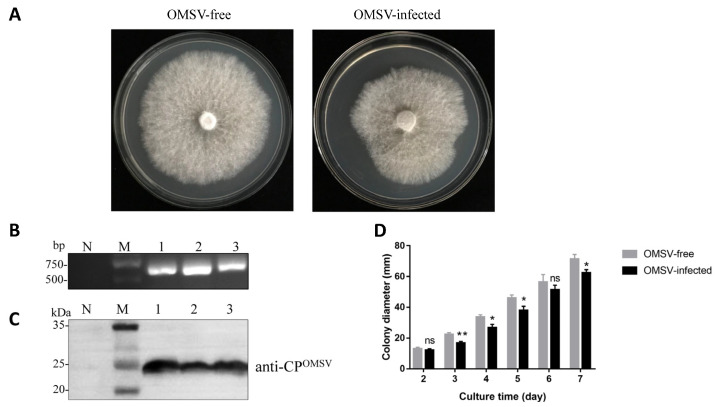
Morphology of isogenic strains of *Pleurotus pulmonarius* after 7 days of incubation. (**A**) Healthy *P. pulmonarius* with regular morphology. OMSV-infected stain showing some extreme sectoring. (**B**) RT-PCR detection of OMSV used to verify the virus transmission. N, negative control, the healthy *P. pulmonarius* strain; M, DNA Marker2000. (**C**) A Western blot of OMSV used to verify the virus presence. Lane M, molecular weights of protein marker. (**D**) The growth rate of OMSV-free and OMSV-infected isogenic strains of *P. pulmonarius*. The independent sample *t*-test was performed between the OMSV-free and OMSV-infected isogenic strains (ns, not significant; * *p* < 0.05; ** *p* < 0.01).

To investigate if OMSV infection affects *P. pulmonarius* mycelial growth, OMSV-free and OMSV-infected isogenic strains were cultured on PDA plates (Figure 3A). The OMSV infection in the *P. pulmonarius* mycelia was detected using RT-PCR (Figure 3B) and Western blot (Figure 3C). Following the seven-day incubation, the virus-infected strain exhibited a reduced mycelial growth rate compared with the virus-free strain (Figure 3A). To confirm this finding, the mycelial growth rate was monitored by measuring the colony diameter every other day for seven days. The OMSV-infected strain grew at approximately 0.89 times slower rate than the OMSV-free strain at 7 DPI (days post-inoculation) (Figure 3D).

### 2.3. Effects of OMSV Infection on the Phenotype of the Fruiting Bodies in P. pulmonarius

**Figure 4:**
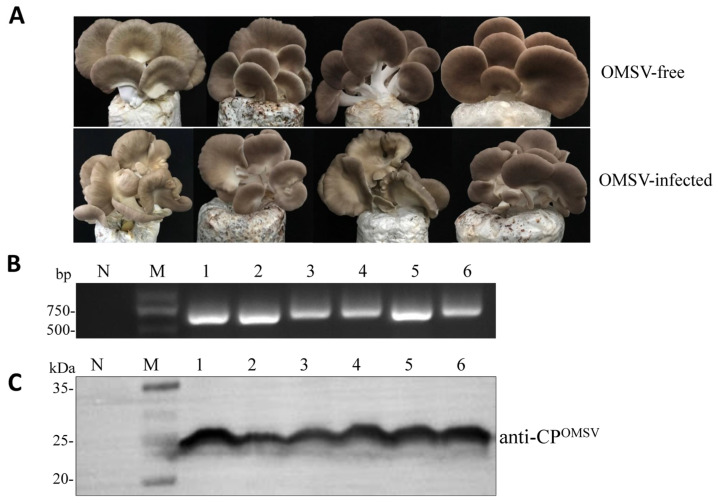
The performance of fruiting bodies affected by OMSV infection in *Pleurotus pulmonarius*. (**A**) Morphological observation and comparison of fruiting bodies of the OMSV-free and OMSV-infected strains. RT-PCR (**B**) and Western blot (**C**) detection of OMSV in fruiting bodies of both strains. Numbers 1–6 represent six biological replicates. The healthy *P. pulmonarius* sample was used as negative control (N). Lane M (**B**), DNA Marker2000. Lane M (**C**), molecular weights of protein marker.

To examine if OMSV infection influenced *P. pulmonarius* fruiting body phenotype, the OMSV-free and OMSV-infected isogenic strains were subjected to cultivation experiments, and the average cultivation period from inoculation to harvest of the first flush mushrooms was calculated. There was no significant difference in the period from inoculation to harvest between OMSV-free and OMSV-infected strain (Table 1). We also observed the morphology of the fruiting bodies and found that the OMSV-infected strain produced abnormal mushrooms with a twisted stem or irregular cap edges (Figure 4A). In contrast, the OMSV-free strain grew normal mushrooms without any detectable symptoms (Figure 4A). To confirm the presence of OMSV in the fruiting bodies, the deformed fruiting bodies were randomly collected and screened with RT-PCR (Figure 4B). Sequence progeny for OMSV in the fruiting bodies was determined by amplicon sequencing. Furthermore, a high accumulation of OMSV-CP in the fruiting bodies of the OMSV-infected *P. pulmonarius* strain was detected with Western blot analyses (Figure 4C). In contrast, no OMSV-CP accumulation was detected in the OMSV-free *P. pulmonarius* strain (Figure 4C).

### 2.4. OMSV Infection Reduced Fruiting Body Yield in P. pulmonarius

In order to further investigate the possible effects of OMSV infection on the yield of the fruiting bodies of *P. pulmonarius*, the first and second flushes of the fruiting bodies were harvested to calculate the average yield (Table 1). By the first flush, *P. pulmonarius* yield exhibited a significant difference between OMSV-free and OMSV-infected strains. The cultivated fruit body average yield of the OMSV-free strain was 173.9 g/bag, which was 1.42-fold higher than that of the OMSV-infected strain (122.76 g/bag). By the second flush, the OMSV-infected strain had an average yield of 105.42 g/bag, which was 0.77-fold lower than that of the OMSV-free strain (136.62 g/bag) (Table 1). These data suggest that the OMSV infection reduced the *P. pulmonarius* fruiting body yield.

### 2.5. Carboxymethyl Cellulase and Laccase Activity in P. pulmonarius Mycelia

**Figure 5:**
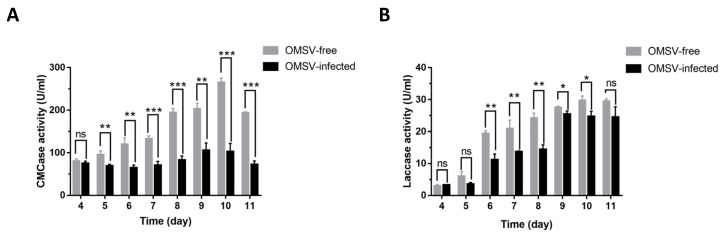
Enzyme activity of OMSV-free and OMSV-infected *Pleurotus pulmonarius* strains during mycelial growth. (**A**) CMCase activity; (**B**) laccase activity. Numbers 4–11 indicate days of liquid mycelial growth. Asterisks indicate significance determined by independent sample *t*-test, * *p* indicates significant difference at <0.05, ** indicates significant difference at *p* < 0.01, and *** indicates significant difference at *p* < 0.001; ns, not significant.

As a member of white-rot fungi, *P. pulmonarius* uses extracellular enzymes to degrade lignocellulose providing nutrients for mycelial growth. The activity of carboxymethyl cellulase (CMCase) and laccase during *P. pulmonarius* mycelial growth was therefore measured (Figure 5). CMCase activity in the OMSV-free strain peaked on the tenth day and the ninth day in the OMSV-infected strain. The OMSV-infected strain demonstrated a significantly lower CMCase activity than that in the healthy strain (Figure 5A). Laccase activity of the OMSV-infected strain was low for the first eight days but then suddenly increased on the ninth day. General trends are observed that are consistent with what is expected from the OMSV-free strain (Figure 5B).

## 3. Discussion

The OMSV China strain (OMSV-Ch) has been previously reported to have the ability to be horizontally transferred from an infected *P. ostreatus* strain to a virus-cured strain via mycelia [30]. This discovery ignited our curiosity about whether OMSV could infect other *Pleurotus* species. In the current study, there were obvious barrage lines between two strains during the co-culturing of *P. ostreatus* and other *Pleurotus* species. Interestingly, OMSV was able to transfer from the original host to the recipient strain of *P. pulmonarius*. These results demonstrated that the OMSV is therefore capable of infecting new hosts across the species barrier. Similarly, the Cryphonectria naterciae fusagravirus 1 (CnFGV1) can be horizontally transferred from *C. naterciae* to *Cryphonectria carpinicola* and *Cryphonectria radicalis* by co-cultivation. Cryphonectria nitschkei chrysovirus 1 (CnCV1) infects three different *Cryphonectria* species by co-culturing, virion transfection, and protoplast fusion [37]. Cryphonectria carpinicola fusagravirus 1 (CcFGV1) in *C. carpinicola* could be introduced into the *C. carpinicola* and *C. parasitica* strains via virion transfection [38]. Moreover, the OMSV failed to transfer to *P. eryngii*, *P. citrinopileatus*, *P. nebrodensis*, and *P. salmoneostramineus*. The failed OMSV transmission to these four species may be due to the limited number of strains in the test. Furthermore, the possibility that a strong transmissibility barrier between the two test strains exists cannot be discounted. According to the phylogenetic studies of species in the *Pleurotus* genus, *P. ostreatus* clusters with *P. pulmonarius,* and they have a closer relationship than with other species (*P. eryngii*, *P. citrinopileatus*, *P. nebrodensis*, or *P. salmoneostramineus*) [39], suggesting a stronger interspecies transmission barrier between the two non-transmissible species.

Although most mycovirus infections are latent with no detectable symptoms, several mycoviruses cause deleterious effects in the host [22,40,41,42]. Infection by several mycoviruses has been previously reported to cause phenotypic aberrations and yield loss in cultivated mushrooms including *P. ostreatus*, *A. bisporus*, and *L. edodes* [20,43,44]. Previously, OMSV was reported to be a causative agent of the difficult-to-control oyster mushroom die-back disease [5]. In our previous study, an OMSV China strain was identified in deformed fruiting bodies of the *P. ostreatus* 8129 strain, and the OMSV-Ch-cured isogenic *P. ostreatus* strain was obtained [30]. However, there was no clear evidence to confirm the etiology of OMSV. To elucidate this, the OMSV-Ch was back-introduced into the virus-cured subculture via co-culture [30]. Recent studies have shown that the OMSV-Ch-newly infected strain demonstrated inhibited mycelial growth, caused malformation symptoms, and reduced the fruiting body yield of the edible mushroom *P. ostreatus* 8129 strain (unpublished data). In the current study, the effect of OMSV infection on *P. pulmonarius* was also investigated. Similarly, the OMSV-infected *P. pulmonarius* strain exhibited phenotypic changes and yield loss of the fruiting bodies, suggesting that OMSV has the potential to be the major pathogen causing yield loss in *P. pulmonarius*. As extracellular enzyme activity consequently enhances mycelial growth and fruiting body production, they are crucial to obtaining the highest yield of cultivated mushrooms. As reported, mycovirus infection reduces the growth of their fungal host associated with a decrease in the activity of laccase and cell wall degrading enzymes [45,46,47]. The infection of the dsRNA PoV in *P. ostreatus* affects spawn growth and fruiting body formation by directly reducing gene expression and then impairing the activity of some extracellular enzymes [43]. In the present study, OMSV infection reduced CMCase and laccase activity in the *P. pulmonarius* strain. This may therefore be the cause of mycelial growth inhibition and fruiting body malformation. However, few studies have focused on the interaction between the mycovirus and its host, specifically in edible fungi. Therefore, these results combined with transcriptome sequencing data that explore other host factors and pathways have the potential to further elucidate the underlying molecular mechanisms of OMSV pathogenicity.

In summary, cross-species transmission of OMSV between *P. ostreatus* and *Pleurotus* species was investigated. OMSV was transmitted horizontally to the *P. pulmonarius* XH2208 strain via hyphal contact. Additionally, OMSV-induced traits in *P. pulmonarius* included inhibited mycelial growth, induced dysmorphic symptoms in fruiting bodies, reduced mushroom yield, and decreased enzymatic activity of CMCase and laccase. To the best of our knowledge, this is the first report detailing OMSV cross-species transmission in edible fungi. The results of this study can provide a better understanding of the potential threat posed by OMSV to *P. pulmonarius* and other *Pleurotus* species.

## 4. Materials and Methods

### 4.1. Horizontal Transmission of OMSV

To investigate whether OMSV can be transmitted across species, we conducted horizontal transmission experiments. *P. ostreatus* strain 8129 (OMSV-infected; donor) and *P. eryngii* strain C1021, *P. citrinopileatus* strain Y055, *P. nebrodensis* strain BN18, *P. pulmonarius* strain XH2208, and *P. salmoneostramineus* strain TH20901 (OMSV-free; recipient) were co-cultivated separately on PDA at 25 °C for 6–8 days, and all strains were deposited in the Fungarium of the Ludong University. Following contact, the mycelial agar plugs from two inocula of the recipient were sub-cultured onto PDA medium. Horizontal transmission of OMSV was detected by RT-PCR amplification using special OMSV primers.

### 4.2. RNA Extraction and Reverse Transcription PCR

About 0.1 g of fresh mycelium was collected and ground in liquid nitrogen. Total RNAs were extracted using RNA Easy Fast Plant Tissue Kit (Tiangen, Beijing, China). For the RT reaction, 10 μL of RT master mixture containing 6 μL ddH_2_O, 3 μL 5× RT-PCR buffer, 1 μL dNTP mix, 0.5 μL reverse primer (OMSV-R), 0.25 μL M-MLV reverse transcriptase, and 0.25 μL RNase Inhibitor was added to 5 μL RNA. Reverse transcription was carried out at 37 °C for 90 min. The final volume of PCR was 15 µL, containing 0.2 µM each primer (OMSV-F/OMSV-R), 1× *Taq* PCR MasterMix II (Tiangen, Beijing, China), and 2 µL cDNA template. PCR cycling was performed as follows: 95 °C for 3 min, then 34 cycles of amplification with 95 °C for 30 s, 53 °C for 50 s, and 72 °C for 30 s, and a final extension at 72 °C for 10 min. For multiplex PCR, the program was set as follows: 95 °C for 5 min, followed by 30 cycles of 3 steps including 95 °C for 30 s, 62 °C for 30 s, 72 °C for 60 s, and a final extension at 72 °C for 10 min [48]. Amplification products were electrophoresed at 125 V for 40 min in 1% agarose gel.

### 4.3. Prokaryotic Expression and Preparation of Polyclonal Antiserum

The OMSV-CP gene was amplified by PCR with primers CPNdF/CPSaR (Table S1) and ligated with a prokaryotic expression vector pDB.His.MBP to obtain pDB.His.MBP-OMSV-CP. The recombinant plasmid was transformed into *Escherichia coli* BL21 (DE3) and induced by 0.1 mM isopropyl-beta-D-thiogalactopyranoside (IPTG). Cultures were grown at 16 °C with shaking for 16 h. Then, the cells were collected by centrifugation at 4000× *g* for 10 min, resuspended in high-salt buffer (20 mM Tris-HCl, 500 mM NaCl, pH 8.0), and disrupted by sonication. The supernatants were applied to a Ni-affinity column, and the recombinant protein was eluted with elution buffer (20 mM Tris-HCl, 150 mM NaCl, 100 mM imidazole, pH 8.0). The purified protein was analyzed through SDS-PAGE electrophoresis.

After purification, the recombinant protein was concentrated to 2 mg mL^−1^, and 5 mg protein was used to immunize a rabbit for preparation of the polyclonal antiserum in Beijing Huada Protein Research & Development Center Co., Ltd. (Beijing, China).

### 4.4. Protein Extraction and Western Blot

The harvested mycelia were ground to powder in liquid nitrogen and then mixed with 2× SDS loading buffer (100 mM Tris-HCl, 4% SDS, 0.2% bromophenol blue, and 20% glycerol). The mixture was boiled at 100 °C for 10 min and centrifuged at 12,000 rpm for 10 min. Protein samples were separated by 12.5% SDS-PAGE and then transferred to a polyvinylidene fluoride membrane. To detect the His.MBP-OMSV-CP expression, the membrane was incubated with His-tag rabbit polyclonal antibody followed by horseradish peroxidase (HRP)-labeled goat anti-rabbit secondary antibody (Shanghai Epizyme Biomedical Technology, Shanghai, China). For detection of OMSV, the membrane was incubated with rabbit anti-OMSV CP polyclonal antiserum followed by HRP-labeled goat anti-rabbit secondary antibody (Shanghai Epizyme Biomedical Technology). The antibody–antigen interactions were visualized by using Omni-ECL™ Pico Light Chemiluminescence Kit (Shanghai Epizyme Biomedical Technology) according to the manufacturer’s instructions.

### 4.5. Mycelial Growth Rate Measurement

To evaluate the effect of OMSV on colony morphology, OMSV-free and OMSV-infected strains inoculated on the center of the PDA medium in a Petri dish with a diameter of 7.5 mm were incubated at 25 °C in the dark. After inoculation, mycelial radial growth was measured from the middle of the fungal inoculum towards the side of the plate using a ruler. The measurements were performed daily for seven days. Meanwhile, the mycelial growth rate (mm/day) was calculated. Three biological replicates were assayed for each sample.

### 4.6. Cultivation of P. pulmonarius

The active mycelium was cultivated on PDA. A sterilized cylindrical cutter was used to knock off 7.5 mm of PDA plate culture for the inoculum. The inocula were cultivated in potato dextrose broth (PDB) in the dark at 25 °C in a 500 mL Erlenmeyer flask and shaken for 8 days at 150 rpm. The basic culture substrates contained cotton-seed hull (80%), wheat bran (18%), and lime powder (2%). Before blending the substrates, cotton-seed hulls were soaked in clean tap water and left overnight at room temperature. The moisture content was verified by squeezing the substrate in the palm until water was no longer observed. Blended substrates, weighing 1.5 kg, were then filled into polypropylene bags and autoclaved at 121 °C for 2 h followed by cooling at room temperature. After cooling, each bag was inoculated with 25 mL of mycelia of *P. pulmonarius* grown in PDB. The top opening of each bag was stuffed with cotton and placed in a dark spawn running room at a temperature of 22–25 °C. When the bags were fully colonized by mycelia, they were moved to a light room for fructification. Proper ventilation and 85–90% relative humidity of the growth room were maintained by opening the door and using humidifiers. The temperature of the fruiting room was maintained at 20–22 °C with an air conditioner. The bags were then opened by gently pulling using a knife. Harvesting was performed when the fruiting bodies were well-developed, 7 days following primordial initiation. During the cropping period, the mushroom flushes were harvested twice, and a total of 80 bags of *P. pulmonarius* yield were measured. The SPSS (version 20) was used for statistical analysis. The independent sample *t*-test was used to analyze the significance of the variation. Values were considered significant when the *p*-value < 0.05. The results were presented as mean ± standard deviation.

### 4.7. Measurement of Enzyme Activity

The activity of CMCase was determined using the DNS method at 540 nm. One unit (U) of enzyme was defined as the amount that releases 1 µmol of CMCase equivalents per minute [49]. We used 2,2′-azino-bis (3-ethylbenzthiazoline-6-sulphonate) (ABTS) to determine the activity of laccase. The reaction mixture contained 0.3 mL of 10 mM ABTS, 3.5 mL of 0.1 mol/L NaAc-HAc buffer (pH 4.6), and 0.2 mL of the supernatant liquid. Oxidation of ABTS was followed by measuring the increase by 420 nm (ε = 36,000 M^−1^ cm^−1^). The catalytic conversion of 1 μmol ABTS within 1 min was regarded as one unit of enzyme activity [50].

## Figures and Tables

**Table 1:** The influence of OMSV infection on *Pleurotus pulmonarius* measured in the cultivation test.

Strains	Period from Inoculation to Harvest (day)	1st Flush Yield (g/bag)	2nd Flush Yield (g/bag)
OMSV-free	29.66 ± 1.52	173.9 ± 12.33	136.62 ± 10.19
OMSV-infected	32.00 ± 1.00	122.76 ± 6.58 **	105.42 ± 11.16 *

* *p* < 0.05 indicates that differences are statistically significant; ** indicates a significant difference (*p* < 0.01) between OMSV-free and OMSV-infected strains.

## Data Availability

The data presented in this study are available within the article.

## Supplementary Materials

The following supporting information can be downloaded at https://www.mdpi.com/article/10.3390/ijms241310584/s1.
