# Supplementary material for: Oyster Mushroom Spherical Virus Crosses the Species Barrier and Is Pathogenic to a New Host Pleurotus pulmonarius
Source: Int J Mol Sci. 2023 Jun 24;24(13):10584. doi: 10.3390/ijms241310584 (PMC10341456; doi:10.3390/ijms241310584)
Supplement: Supplementary file 1 [file ijms-24-10584-s001.zip › ijms-2391083-supplementary.pdf]

Table S1. Sequences of primers used in this study.

Primer	Sequence (5′-3′)	Note [a]
OMSV-F	ACCCCCCCAGGATCTCAAGCTTC	RT-PCR detection for OMSV [48]
OMSV-R	GAGATGTAGACRTTGAAAGC	
OMIV-F	AACATTGTTGATCACGCTCT	RT-PCR detection for OMIV [48]
OMIV-R	GGCTTCAGAATAAAGATTGT	
POSV-F	ATCWCATGGCTATCAACCTA	RT-PCR detection for POSV [48]
POSV-R	AGCTGAATTATCGTCACCCA	
PoV1-F	AAACTCGAAGAGTTCCTTTC	RT-PCR detection for PoV1 [48]
PoV1-R	GCGCGTGGGCCACGTTCGGG	
CPNdF	CATATGATGTCTCTTGCTACCCCC	Used for construction of the prokaryotic expression vector pDB.His.MBP-OMSV-CP
CPSaR	GTCGACGATGACGCCGTACCCG	

[a] OMSV, oyster mushroom spherical virus; OMIV, oyster mushroom isomeric virus; POSV, Pleurotus ostreatus spherical virus; PoV1, Pleurotus ostreatus virus 1.

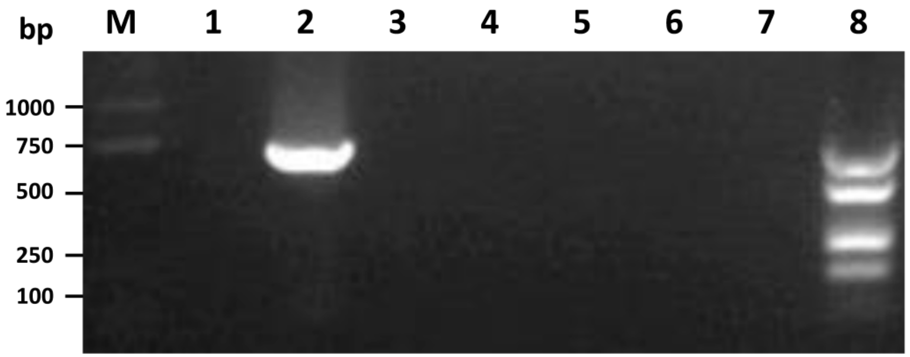

Figure S1. Viruses detection of the donor and recipient strains by multiplex RT-PCR before co-cultivation. Lane 1, negative control, the OMSV-free *P. ostreatus* strain. Lane 2, OMSV-infected *P. ostreatus* 8129 strain (donor). Lanes 3-7 represent *P. eryngii* strain C1021, *P. citrinopileatus* strain Y055, *P. nebrodensis* strain BN18, *P. pulmonarius* strain XH2208, and *P. salmoneostramineus* strain TH20901, respectively. Lane 8, the plasmids containing genome sequences of OMSV, PoV1, POSV, and OMIV as positive control [48]. Lane M: GL DNA Marker2000.
